# Pyrexia of Unknown Origin (PUO) in South Asia: A Systematic Review of Diagnostic Gaps and Management Strategies

**DOI:** 10.21203/rs.3.rs-8713979/v1

**Published:** 2026-03-18

**Authors:** Birendra Gupta, Natalia Blanco, Chandramani Wagle, Nikita Acharya, Jyoti Takanche, Rajeev Shrestha, Emilie Ludeman, Tracy Hazen, Man Charurat

**Affiliations:** Institute of Human Virology, University of Maryland School of Medicine; Institute of Human Virology, University of Maryland School of Medicine; Global Clinical Research; Global Clinical Research; Center for Infectious Disease Research and Surveillance, Dhulikhel Hospital Kathmandu University Hospital; Center for Infectious Disease Research and Surveillance, Dhulikhel Hospital Kathmandu University Hospital; Health Sciences and Human Services Library University of Maryland; Center for Advanced Microbiome Research and Innovation, Institute for Genome Sciences, University of Maryland School of Medicine; Institute of Human Virology, University of Maryland School of Medicine

**Keywords:** PUO, FUO, Diagnostic Gap, Etiological Spectrum, Management Strategy, South Asia

## Abstract

**Background:**

Pyrexia of Unknown Origin (PUO) represents a persistent diagnostic challenge in clinical practice. In South Asian regions, this challenge is compounded by a high burden of endemic infections, resource constraints, and varied clinical practices. This systematic review aimed to synthesize the available evidence on the etiology, diagnostic approaches, management, and systemic barriers related to PUO in the region to inform improved clinical guidelines and health policies.

**Methods:**

A systematic literature search was conducted in major electronic databases following PRISMA 2020 guidelines and registered prospectively in PROSPERO (CRD420251170142). Observational studies and case series reporting PUO among all populations in South Asian countries were included. Dual independent screening, data extraction, and quality assessment were performed. Risk of bias was evaluated using the Joanna Briggs Institute (JBI) tools for observational studies and QUADAS-2 for diagnostic accuracy studies. Findings were synthesized narratively due to substantial clinical and methodological heterogeneity.

**Results:**

Thirty-seven studies were included, with infectious diseases predominating, most commonly tuberculosis, enteric fever, and scrub typhus. Non-infectious inflammatory diseases and malignancies accounted for significant minorities. Diagnostic yield varied substantially across studies, with higher yields reported in settings with greater access to advanced diagnostic modalities. Key systemic barriers included limited laboratory and imaging capacity, high rates of empirical antimicrobial use, absence of standardized diagnostic protocols, and patient-related factors like late presentation and cost. Management was largely empirical, with third-generation cephalosporins and doxycycline commonly used. A striking geographical imbalance in evidence was noted, with most studies originating from India.

**Conclusions:**

PUO in South Asia is characterized by a heterogeneous etiological spectrum, persistent diagnostic uncertainty, and widespread reliance on empirical management. The findings indicate that it is necessary to develop standardized, context-appropriate PUO definitions and diagnostic algorithms, improve access to targeted investigations, and strengthen antimicrobial stewardship to enhance patient care in resource-limited settings. Future research must address the significant geographical gaps in evidence and focus on implementing standardized approaches across the region.

## Background

Pyrexia of Unknown Origin (PUO) remains one of the most threatening diagnostic challenges in modern clinical practice [1]. PUO also referred to as Fever of Unknown Origin (FUO) in some literature, was first defined by Petersdorf and Beeson in 1961, characterizing it as a body temperature above 38.3°C (101°F) on several occasions, lasting for more than three weeks, with no diagnosis reached after one week of intensive hospital evaluation [2]. Despite six decades of advances in serological testing, molecular diagnostics, and imaging technologies, PUO continues to confound clinicians globally, with 17–35% of cases attributed to infections, 24–36% to inflammatory causes, 10–20% to neoplastic conditions, and up to 51% remaining undiagnosed even after extensive evaluation [3–5]. The complexity of diagnosing PUO arises from its heterogeneous etiology. More than 200 potential causes have been identified, including infectious diseases, malignancies, autoimmune disorders, and miscellaneous conditions [6].

The etiological spectrum of PUO is geographically and economically heterogeneous, shaped by local disease prevalence, healthcare resources, and investigative protocols [7, 8]. Globally, infections, non-infectious inflammatory diseases (NIID), and neoplasms constitute the primary diagnostic categories, but their relative prevalence varies significantly [7]. Regional meta-analyses reveal distinct patterns; for instance, the Western Pacific region reports the highest pooled estimates for NIIDs (27%), while the Eastern Mediterranean region shows the highest prevalence of malignancy-associated PUO (25%) [9]. This geographic determinism highlights that a “one-size-fits-all” diagnostic algorithm is ineffective and that clinical evaluation must be informed by local epidemiological data and resource availability [9].

South Asia, comprising India, Pakistan, Bangladesh, Nepal, Sri Lanka, Bhutan, Maldives, and Afghanistan, bears a substantial burden of endemic infections such as tuberculosis, typhoid fever, malaria, and dengue, alongside non-infectious etiologies including malignancies and autoimmune disorders. This complex epidemiological landscape poses unique and significant challenges in the evaluation of PUO [10, 11]. The diagnostic process is frequently complicated by overlapping clinical presentations, high patient volumes in tertiary care centers, and systemic constraints including limited access to advanced diagnostics (e.g., molecular assays, automated blood culture systems, advanced imaging such as CT/MRI, and histopathology) and significant out-of-pocket healthcare costs [5, 12]. A study from Sri Lanka illustrates this burden: despite extensive workup, 35% of PUO patients remained undiagnosed, infections (primarily extrapulmonary tuberculosis) were the leading cause (72.3%), and the mean direct cost of care per patient was substantial (USD 467.79), with investigations alone constituting nearly half of this expense [5]. These factors frequently result in undiagnosed cases or delayed diagnoses, leading to prolonged hospitalization and inappropriate empirical use of antimicrobials and corticosteroids [13, 14]. Such practices may mask underlying pathologies and exacerbate poor clinical outcomes, underscoring the urgent need for context-appropriate, evidence-based guidelines [5].

The importance of conducting this systematic review lies in consolidating evidence on the etiological spectrum, diagnostic approaches, barriers, and management strategies specific to South Asia. No comprehensive systematic review has yet synthesized data across the region, despite calls for region-specific analyses to address geographic variations in PUO etiology [15, 16]. Specifically, the objectives are to (i) describe the etiological spectrum of PUO and the diagnostic approaches employed across South Asian countries; (ii) to identify diagnostic challenges and barriers, including institutional, systemic, and resource-related constraints; (iii) to summarize reported management strategies, including empirical treatments and follow-up practices; and (iv) to generate evidence-informed recommendations to improve PUO diagnosis and management in the region. To ensure a rigorous and transparent approach, this review is structured according to the Population, Intervention, Comparator, Outcome, and Study Design (PICOS) framework. The review includes patients of all age groups diagnosed with PUO (Population), evaluates diagnostic modalities and management strategies (Interventions), and synthesizes evidence on etiological distribution and diagnostic yield (Outcomes) within the South Asian geographical context.

## Methods

### Protocol and Registration

This systematic review was conducted in accordance with the Preferred Reporting Items for Systematic Reviews and Meta-Analyses (PRISMA) 2020 guidelines. The protocol was prospectively registered with the International Prospective Register of Systematic Reviews (PROSPERO; Registration Number: CRD420251170142) and was published on October 30, 2025.

### Eligibility Criteria

Studies were selected according to predefined inclusion and exclusion criteria based on population, study design, setting, and outcomes of interest.

Studies were selected based on the following inclusion criteria:

- Studies report patients of any age group with PUO or FUO (considered synonymous), based on any accepted classical definition (e.g., Petersdorf & Beeson [2]; Durack & Street [17]), a modified definition, or an explicitly stated clinical definition.
- Peer-reviewed original research with observational designs (cross-sectional, cohort, or case-control studies), hospital audits, or descriptive case series with ≥5 patients.
- Studies conducted within South Asian countries (Afghanistan, Bangladesh, Bhutan, India, Maldives, Nepal, Pakistan, and Sri Lanka).
- Reported at least one of the following outcomes: etiological spectrum, diagnostic methods and yield, management strategies, or barriers to diagnosis.
- Studies in which patients initially presented with acute undifferentiated fever and were subsequently followed to meet PUO/FUO criteria were included, provided that diagnostic evaluation and outcomes were reported within a PUO/FUO framework.
- Articles published in English from January 2000 to November 2025 with accessible full text. Studies predating 2000 were excluded to ensure relevance to contemporary diagnostic capabilities and epidemiological patterns.

Exclusion criteria were:

1. Reviews, editorials, commentaries, conference abstracts, or case reports/series with fewer than five cases.
2. Studies that exclusively focus on a single febrile disease without contextualizing it as PUO/FUO.
3. Research studies that often lack sufficient data for meaningful extraction or synthesis.

### Information Sources and Search Strategy

A comprehensive literature search was conducted across PubMed/MEDLINE, Embase, Scopus, and the Web of Science. In addition, regional databases, including the Index Medicus for the Southeast Asia Region (IMSEAR), were searched to capture locally published studies. To identify additional relevant literature, reference lists of included studies and relevant review articles were hand-searched (backward citation tracking), and forward citation tracking was performed using Google Scholar and Scopus. The search was updated on 5 December 2025 to capture any newly published literature.

Search strategies were developed in consultation with a medical librarian and combined controlled vocabulary terms (e.g., Medical Subject Headings [MeSH]) with free-text keywords related to PUO/FUO and South Asian countries. The strategy combined terms for PUO (e.g., “Pyrexia of Unknown Origin,” “Fever of Unknown Origin,” PUO, and FUO) with individual South Asian country names (India, Nepal, Pakistan, Bangladesh, Sri Lanka, Bhutan, Maldives, and Afghanistan). Synonyms and regional variations (e.g., “undifferentiated fever”) were incorporated to maximize retrieval. No restrictions were applied based on outcomes or study design beyond publication date and language. Grey literature sources (e.g., conference proceedings, theses, and preprints) were not searched, and no automation tools were used during the search process. A summary of the search strategy is presented in Box 1, and the full electronic search strategies for all databases are provided in Additional File 1.

### Study Selection Process

All retrieved records were imported into Covidence systematic review software for management and screening. Covidence is a web-based collaboration software platform that streamlines the production of systematic and other literature reviews [18]. After removal of duplicates, two reviewers independently screened titles and abstracts for eligibility. Full texts of potentially relevant articles were subsequently assessed independently by the same reviewers.

Discrepancies at any stage were resolved through discussion and, when necessary, by consultation with a third reviewer. No automation or machine-learning tools were used beyond Covidence’s standard deduplication functions. The study selection process is summarized in the PRISMA flow diagram presented in the Results section.

### Data Collection Process and Data Items

Data were extracted independently by two reviewers using a customized extraction form in Covidence. Extracted items included study characteristics (author, year, country, setting, design, sample size), participant demographics (age), PUO definition and criteria, diagnostic tools and their yield, reported etiologies and proportional distribution, time to diagnosis, unresolved cases, management strategies (including empirical therapies and follow-up), and identified barriers or challenges. Any discrepancies were resolved by consensus or third-reviewer arbitration. Where data were unclear or incomplete, information was inferred from the published text when possible; study authors were not contacted for clarification. Missing information was noted as “not reported.”

### Study Risk of Bias Assessment

Risk of bias in included studies was assessed independently by two reviewers using tools appropriate to study design: the Joanna Briggs Institute (JBI) Critical Appraisal Checklists for observational studies (e.g., cohort, cross-sectional, case series) [19, 20], QUADAS-2 for any diagnostic accuracy components [21]. Each item was rated as *Yes*, *No*, *Unclear*, or *Not Applicable*. Any disagreements in quality measures were resolved through discussion. Risk-of-bias assessments informed interpretation but were not used as exclusion criteria.

### Effect Measures

Given the descriptive nature of this systematic review, which primarily aimed to characterize the etiological spectrum of PUO/FUO, diagnostic approaches, management strategies, and reported barriers, formal comparative effect measures (such as risk ratios or mean differences) were not applicable.

Outcomes were summarized using proportions and percentages, including the proportion of PUO cases attributed to specific etiological categories, the proportion of diagnosed versus undiagnosed cases, and reported mortality rates where available. For studies contributing quantitative data, these measures were presented at the individual study level and, where appropriate, synthesized descriptively. No thresholds for effect size interpretation were prespecified, as the review did not aim to evaluate the magnitude of comparative effects between interventions or exposures.

### Synthesis Methods

Studies meeting the inclusion criteria were synthesized according to reported outcomes and methodological characteristics. Given the substantial clinical, methodological, and epidemiological heterogeneity across studies including variation in PUO/FUO definitions, diagnostic capacity, study settings, and time periods, a narrative synthesis was undertaken. Findings were organized thematically in line with the review objectives: etiological spectrum, diagnostic approaches and yield, diagnostic challenges and barriers, and management strategies. Individual studies could contribute to multiple thematic syntheses depending on the outcomes reported.

Extracted data were reviewed for consistency and completeness prior to synthesis. Where necessary, proportions were recalculated using reported numerators and denominators to ensure comparability across studies. Missing summary statistics were noted as “not reported” and no imputation was performed. Results were summarized using structured tables presenting study characteristics, population details, diagnostic approaches, etiological distributions, and key outcomes. Narrative findings were summarized in thematic tables. Studies were ordered in tables by country to highlight geographical patterns. Formal meta-analysis was not conducted due to heterogeneity and the predominantly descriptive nature of the available evidence. Instead, heterogeneity was explored qualitatively by comparing findings across study-level characteristics, including country, healthcare setting, study period, and reported diagnostic resources.

### Reporting Biases and Certainty of Evidence

Given the descriptive nature and substantial heterogeneity of the included studies, which precluded quantitative meta-analysis, assessments for reporting biases and the certainty of evidence were conducted at the narrative synthesis level.

### Reporting Biases

The potential for bias due to missing results (e.g., publication bias) was considered qualitatively. This evaluation was based on the comprehensiveness of the search strategy (including regional databases and citation tracking) and an examination of the body of evidence for signs of selective reporting, such as a lack of studies reporting negative or inconclusive diagnostic findings.

### Certainty of Evidence

A formal GRADE assessment was not applicable. Instead, the overall confidence in the synthesized findings was judged narratively by integrating the results of the critical appraisal (risk of bias), the consistency of results across studies and settings, and the directness of the evidence to the review’s context and objectives.

## Results

### Study Selection

**Figure 1:**
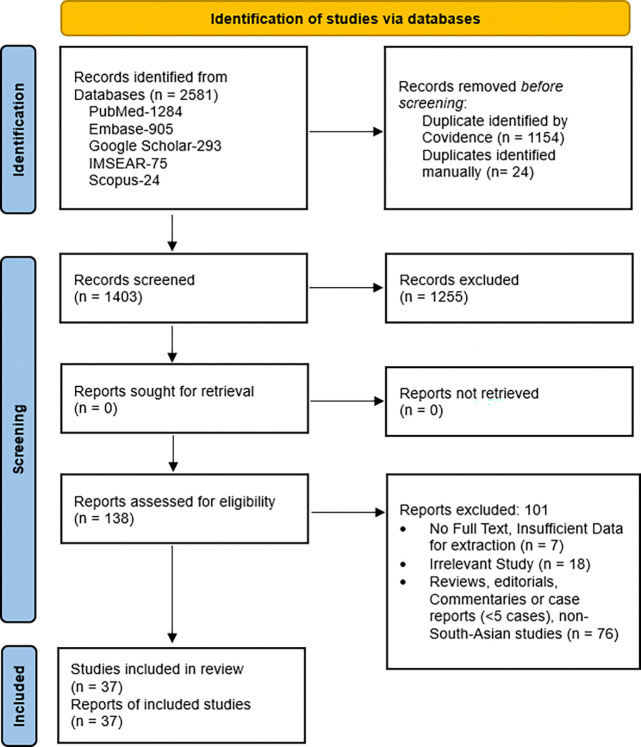
PRISMA flow diagram of literature search and study selection.

The systematic search across five electronic databases (PubMed, Embase, Google scholar, IMSEAR and Scopus) yielded a total of 2,581 records. After automated deduplication in Covidence and additional manual review, 1,178 duplicate records were removed (1,154 automated and 24 manual; Fig. 1). The remaining 1,403 unique records underwent independent title and abstract screening by two reviewers, of which 1,255 were excluded. Full texts were retrieved and assessed for the remaining 138 reports. After full-text evaluation, 101 reports were excluded for the following predefined reasons: Systematic or narrative reviews type, editorials, commentaries, small case reports/series (n = 76), irrelevant study focus (n = 18), insufficient data availability or inaccessible full text (n = 7). Ultimately, 37 studies were included in the qualitative synthesis, with all contributing relevant data for quantitative synthesis. No ongoing studies were identified.

### Study Characteristics

The characteristics of the 37 studies included in this review are summarized in Table 1. Most studies were conducted in India (n = 20, 54.1%), followed by Bangladesh (n = 5, 13.5%) and Nepal (n = 5, 13.5%. Additional studies were from Pakistan (n = 4, 10.8%), Sri Lanka (n = 2, 5.4%), and Bhutan (n = 1, 2.7%), reflecting substantial geographic imbalance in the available evidence across South Asia. Nearly all studies were undertaken in tertiary hospital settings, with no community-based investigations identified. Cross-sectional studies accounted for approximately half of the included studies (n = 18, 48.6%), followed by cohort designs (n = 16, 43.2%), while only one case–control study (2.7%) and one mixed-methods study (2.7%) were included. Sample sizes varied widely, ranging from 24 to 9,739 participants, and study populations included adults (n = 18, 48.6%), children (n = 9, 24.3%), or mixed age groups (n = 9, 24.3%), with one study not reporting population characteristics. The definitions of PUO applied across studies varied significantly; Fifteen (n = 15, 40.5%) studies used classical definitions either Petersdorf & Beeson (prolonged fever ≥ 38.3°C for ≥ 3 weeks with no diagnosis after appropriate inpatient evaluation) or Durack & Street modification, which incorporates modern diagnostic testing and outpatient evaluation frameworks., Ten studies (n = 10, 27%) adopted locally defined criteria, and a notable proportion (n = 12, 32.4%) did not explicitly report the definition used. Locally adapted PUO definitions exhibited variability primarily in the required duration of fever (ranging from ≥ 7 days to ≥ 3 weeks), the clinical setting of evaluation (inpatient-only versus combined outpatient–inpatient assessment), and the extent of diagnostic investigations mandated before classification as PUO. This heterogeneity in case definitions likely influenced study populations, diagnostic intensity, and reported etiological distributions, thereby limiting direct comparison of diagnostic yield and outcome patterns across studies (Table 1).

### Risk of Bias in Included Studies

Among the 37 studies included, the majority were judged to have a moderate risk of bias, reflecting common methodological limitations across the evidence base. Two cohort studies were assessed as having a low risk of bias [37, 50], while a smaller number of studies were rated as having a high risk of bias[28, 32, 46, 49] (Table 2).

Across studies, frequently reported concerns included lack of identification or adjustment for confounding factors, single-center and hospital-based designs, and variability or insufficient detail in diagnostic confirmation methods. Several cohort studies reported unclear or incomplete follow-up, while some cross-sectional studies relied on clinical or presumptive diagnoses without consistent laboratory confirmation. Studies assessed as high risk of bias were primarily affected by outcome misclassification, non-representative sampling, self-reported outcomes, or substantial exclusions or attrition. Specifically, misclassification was commonly related to the use of therapeutic trials (particularly anti-tubercular therapy) as a basis for etiological diagnosis, reliance on syndromic or “presumed” diagnoses without microbiological or histopathological confirmation, and incomplete diagnostic ascertainment due to pending investigations at discharge or limited access to advanced diagnostics.

### Results of Individual Studies

Diagnostic modalities employed and the etiological spectrum reported across included studies for which relevant information was available were summarized in Table 3. The reported proportion of cases in which a final diagnosis was achieved ranged widely from 37% to 100%. The diagnostic workup across studies consistently relied on a combination of basic laboratory tests, serology, and imaging, with a subset utilizing more advanced techniques such as molecular diagnostics, histopathology, or bone marrow biopsy. Infectious diseases were the predominant etiological category in most studies reporting etiological distributions, accounting for more than 50% of diagnosed cases in approximately two-thirds of studies, with reported infectious proportions ranging from ~ 38% to 100% where data were available. Non-infectious inflammatory diseases and malignancies were reported as less common but consistent categories across the region. The proportion of cases that remained undiagnosed despite investigation varied from 0% to 61.9%.

Tuberculosis, particularly extrapulmonary tuberculosis, was the most frequently reported specific etiology across multiple studies, followed by other infectious causes such as enteric fever, scrub typhus, dengue, and respiratory tract infections. Considerable variability was observed in both diagnostic yield and etiological distribution across studies, corresponding to differences in diagnostic strategies and reporting practices, as detailed in Table 3. Laboratory evaluations varied across studies and included basic tests (CBC, ESR/CRP, liver/renal function), serology (pathogen-specific antibody or antigen assays), imaging (X-ray, ultrasound, CT, MRI), histopathology (biopsy), hematology (peripheral smear or bone marrow), and molecular tests (PCR where available).

### Results of Syntheses

#### Diagnostic Approaches, Yield, and Challenges

Diagnostic approaches for PUO/FUO in South Asia were predominantly stepwise and resource-adapted, with heavy reliance on basic laboratory tests (complete blood count, erythrocyte sedimentation rate, C-reactive protein), blood cultures, chest X-ray, abdominal ultrasonography, and serological tests for endemic infections (e.g., typhoid, dengue, malaria, scrub typhus). First-line investigations frequently identified infectious causes, particularly tuberculosis (TB). Second- and third-line tests, including contrast-enhanced computed tomography (CECT) of the chest/abdomen/pelvis, bone marrow examination, and biopsies, were crucial for diagnosing malignancies, non-infectious inflammatory diseases (NIIDs), and miscellaneous conditions [5, 28, 37, 45, 57].

Diagnostic yield varied widely. Non-invasive methods established a diagnosis in more than two-thirds of cases in several studies, while invasive procedures (bone marrow aspiration, biopsies, endoscopy, bronchoscopy) were required for the remainder [44, 49, 50]. Advanced imaging (PET-CT) showed high contributory value (up to 90.7%) in selected cohorts when available, but was rarely accessible [36]. Overall diagnostic success ranged from 65–88% in protocol-driven studies, with undiagnosed cases typically remaining in 10–35% of patients. The synthesis of qualitative data revealed pervasive, interconnected systemic challenges that directly impacted diagnostic success. These barriers were categorized into four primary domains, as summarized in Table 4.

Infrastructural and technical limitations were the most frequently cited constraints. Studies from multiple countries, including Nepal [49, 52], India [30, 32], Bangladesh [22], and Sri Lanka [5], reported limited laboratory capacity, a critical lack of advanced imaging (CT, MRI, FDG-PET/CT), and unavailable molecular diagnostics. These shortages necessitated referrals to external laboratories or other specialized centers, resulting in significant diagnostic delays..

Knowledge and protocol-based barriers further complicated the diagnostic process. A predominant theme was the over-reliance on empirical antimicrobial treatment, noted extensively across studies from India [37, 58], Pakistan [56], Nepal [50], and Bangladesh [26]. This practice, often initiated before conclusive testing, was reported to obscure clinical presentations and microbiological results. Furthermore, a near-universal lack of standardized regional or national diagnostic algorithms led to inconsistent and non-reproducible investigative pathways.

Patient-side and systemic barriers significantly influenced healthcare access and diagnostic completion. Late presentation to advanced care facilities and widespread prior antibiotic use from over-the-counter access or presumptive treatment were commonly reported, particularly in studies from Nepal, India, and Pakistan [34, 50, 54]. Financial constraints and high out-of-pocket costs for investigations were identified as critical factors leading to incomplete workups and loss to follow-up [5, 35]. The consequence of these compounded barriers was frequently delayed diagnosis, prolonged hospital stays, and continued empirical treatment overuse, which studies explicitly linked to driving antimicrobial resistance [28, 52].

#### Management Strategies

Management strategies for PUO reported across included studies were largely shaped by diagnostic uncertainty, local epidemiology, and resource availability and ranged from structured protocol-based care to empiric therapeutic approaches. Most studies described management decisions as being closely linked to evolving diagnostic findings rather than predefined treatment pathways. The strategies reported were categorized into three main themes: empirical antimicrobial therapy, diagnostic-therapeutic trials, and specialty referral (Table 5).

The most consistently reported strategy was the initiation of empirical antimicrobial therapy. Third-generation cephalosporins, particularly ceftriaxone and cefixime, were the most frequently cited first-line empirical antibiotics across multiple studies in India, Pakistan, and Nepal [5, 28, 56]. Doxycycline was commonly employed for suspected rickettsial infections like scrub typhus [33, 37, 51]. A notable finding was the use of anti-tubercular therapy (ATT) as both a diagnostic and management approach: in cases where tuberculosis was clinically suspected but not microbiologically confirmed, initiation of ATT and subsequent clinical improvement were used to support the diagnosis [31, 46, 47].

For cases where non-infectious inflammatory diseases (NIIDs) or malignancies were suspected, management involved referral to specialized care units (e.g., rheumatology, oncology) or the initiation of immunosuppressive therapy or chemotherapy [27, 42]. To navigate diagnostic uncertainty, several studies advocated for or implemented a structured, stage-wise diagnostic protocol to rationalize testing and therapy. This approach emphasizes initial epidemiological review and clinical examination to guide targeted investigations, moving away from non-specific “shotgun” testing [29, 31, 56]. The strategic use of advanced imaging (e.g., CT, PET-CT) to guide biopsies was also reported as a key management-enhancing strategy [32, 47].

#### Reporting Biases and Certainty of Evidence

A formal assessment of publication bias using funnel plots and statistical tests was not performed for the primary outcomes. Assessment of reporting bias was limited by the predominance of single-center, descriptive studies with incomplete or inconsistent reporting of diagnostic criteria, management protocols, and follow-up outcomes. Selective outcome reporting was likely, as several studies emphasized etiological yield or treatment response without clearly defining diagnostic standards or accounting for undiagnosed cases. The comprehensive search strategy, which included regional databases and backward citation tracking, was designed to minimize the risk of missing unpublished or non-indexed studies.

Overall, the certainty of evidence was judged to be low to moderate, driven by high risk of bias, lack of standardized outcome ascertainment, and reliance on empirical or therapeutic trials for diagnosis in multiple studies. While the consistency of infectious etiologies across diverse settings strengthens confidence in broad etiological patterns, the precision and comparability of individual diagnostic and management outcomes remain limited.

## Discussion

This systematic review synthesizes evidence from 37 observational studies conducted in South Asia between 2000 and 2025, highlighting persistent diagnostic and management challenges for PUO/FUO in a region characterized by high infectious disease burden, resource constraints, and geographic heterogeneity in research output. The findings confirm that infectious diseases, particularly tuberculosis, remain the predominant cause of PUO in the region, accounting for a substantial majority of diagnosed cases. However, a significant and variable proportion of cases, with a median diagnostic yield of 84%, were attributable to non-infectious inflammatory diseases and malignancies, underscoring the diagnostic complexity. The review identified pervasive systemic challenges, including infrastructural limitations, over-reliance on empirical antimicrobial therapy, and the absence of standardized diagnostic protocols, which collectively contribute to delayed diagnosis and suboptimal patient outcomes. The review highlights a critical “diagnostic-therapeutic paradox” in the region: while advanced tools like PET-CT and molecular diagnostics show high yield in urban centers, the majority of regional management is still driven by syndrome-based empirical therapy and “therapeutic trials,” particularly with anti-tubercular drugs, due to systemic barriers. A striking geographical imbalance in research output was evident, with the majority of studies originating from India, highlighting critical evidence gaps from several South Asian nations such as Bhutan, Afghanistan, and the Maldives.

The etiological spectrum observed in this review aligns with the recognized global pattern where infections predominate in resource-limited settings but contrasts with trends from high-income regions. In Europe and North America, for instance, non-infectious inflammatory diseases (NIIDs) and malignancies often constitute a larger proportion of PUO cases, while undiagnosed cases are frequently reported as the most common single “category” [59, 60]. The continued dominance of infectious etiologies in the present synthesis likely reflects delayed healthcare access, prior empirical antimicrobial exposure, and endemic disease burden, particularly tuberculosis, enteric fever, rickettsial infections, and malaria.

Consistent with prior literature, this review also confirms that structured, stepwise diagnostic algorithms based on potential diagnostic clues outperform non-systematic or empiric approaches in achieving higher diagnostic yields [31, 56, 60]. While advanced modalities such as PET-CT have been shown to improve etiological identification in selected populations [61], their limited availability and variable sensitivity particularly for small-vessel vasculitis and renal pathology restrict their routine applicability in low-resource settings. Importantly, the frequent reliance on therapeutic trials, especially for tuberculosis, mirrors observations from earlier regional studies but remains at odds with contemporary recommendations that caution against empiric treatment without objective confirmation due to risks of misclassification and antimicrobial resistance [62, 63].

The identified diagnostic challenges have direct and profound implications for both clinical outcomes and health systems. The frequent reliance on empirical antimicrobial therapy, while often a practical necessity, creates a cycle of diagnostic obfuscation and fuels antimicrobial resistance (AMR), a critical public health threat already prevalent in the region [64, 65]. Prior antimicrobial exposure, in particular, has been shown to significantly reduce microbiological yield, leading to missed or misclassified infectious etiologies [66]. The lack of standardized diagnostic algorithms and limited access to advanced diagnostics (e.g., CT/MRI, molecular tests, specialized serology) in non-tertiary centers contribute to prolonged hospital stays, increased out-of-pocket expenses for patients, and delayed initiation of correct, targeted therapy [67, 68]. These barriers disproportionately affect vulnerable populations, exacerbating health inequities. Patient- and system-related factors including late presentation, widespread pre-referral antibiotic use, socioeconomic constraints, and poor follow-up were also prominent contributors to diagnostic uncertainty.

These findings carry important implications for PUO management and health policy in South Asia. The consistent success of structured, stage-wise protocols emphasizing clinical clues (potential diagnostic clues/PDCs), repeated examination, basic targeted tests, and judicious invasive procedures (e.g., bone marrow biopsy, CT-guided sampling) demonstrates that high diagnostic yields are achievable even in resource-limited environments [69]. Such approaches reduce unnecessary broad-spectrum empirical therapy, limit antimicrobial resistance emergence, and optimize resource use critical in a region facing rising AMR and strained health systems. Policy efforts should prioritize developing and disseminating region-specific, context-adapted diagnostic algorithms, strengthening primary/secondary-level laboratory capacity (e.g., basic cultures, serology), and promoting antimicrobial stewardship training for clinicians [5, 64, 70]. Addressing patient-side barriers (late presentation, socioeconomic constraints, prior self-medication) through community education and improved healthcare access could further enhance outcomes [71, 72].

This systematic review has several important strengths. It provides the comprehensive synthesis of PUO evidence from South Asia, incorporating 37 observational studies across six countries. Methodological rigor was ensured through adherence to PRISMA 2020 guidelines, prospective PROSPERO registration, dual independent screening and data extraction, use of validated appraisal tools (JBI and QUADAS-2), and a structured narrative synthesis that accounted for substantial heterogeneity. Several limitations warrant consideration. Marked heterogeneity in PUO definitions, diagnostic criteria, and investigative intensity across studies limited comparability and precluded quantitative synthesis. Evidence was geographically skewed, with most studies originating from India, restricting generalizability to underrepresented countries. The predominance of retrospective, single-center, tertiary-hospital studies likely introduced selection bias and may overestimate diagnostic yields achievable in routine or lower-level care settings. Additionally, reliance on published data alone may have resulted in incomplete capture of diagnostic barriers or management strategies.

These findings highlight key priorities for future research. Prospective, multicenter studies using standardized PUO definitions and including primary and secondary care settings are urgently needed, particularly in underrepresented South Asian countries. Development and validation of resource-stratified, region-specific diagnostic algorithms incorporating potential diagnostic clues and cost-effective investigations should be prioritized. Longitudinal evaluations of protocol-driven approaches and antimicrobial stewardship interventions are essential to improve diagnostic accuracy, reduce empirical treatment, and optimize patient outcomes.

## Conclusions

This systematic review synthesizes available evidence on PUO in South Asia and demonstrates that infectious diseases, particularly tuberculosis and other endemic infections, remain the predominant etiologies, while non-infectious inflammatory disorders and malignancies contribute a substantial minority of cases. Diagnostic yield varied widely across studies and was strongly influenced by resource availability, adherence to structured diagnostic protocols, and access to advanced imaging or invasive investigations. Empirical treatment, especially antibiotics and anti-tubercular therapy, was common and often driven by diagnostic constraints, highlighting persistent challenges in standardized PUO evaluation. These findings underscore the urgent need for standardized PUO definitions, region-specific and resource-adapted diagnostic algorithms, strengthened laboratory infrastructure, antimicrobial stewardship, and expanded research in underrepresented countries to improve PUO outcomes and reduce diagnostic uncertainty across South Asia.

## Supplementary Material

Supplementary Files

This is a list of supplementary files associated with this preprint. Click to download.

• AdditionalFile1Searchstrategy2.docx

## Figures and Tables

**Table 1:** Characteristics of included studies

Study ID	Author (Year)	Country	Design	Study Period	Sample Size (n)	Population	PUO Definition	Ref.
1	Haidar (2018)	Bangladesh	Cross-sectional	Jan 2016 - Dec 2017	72	Adults	Locally adapted	[22]
2	Haque (2025)	Bangladesh	Cross-sectional	Jul 2019 - Jun 2020	344	Mixed	Not explicitly stated	[23]
3	Rahimi (2016)	Bangladesh	Cross-sectional	Jul 2012 - Jun 2013	33	Adults	Petersdorf & Beeson	[24]
4	Haque (2025)	Bangladesh	Cohort	Jul 2021 - Jun 2023	80	Adults	Locally adapted	[25]
5	Bhuiyan (2024)	Bangladesh	Cross-sectional	Jan 2021 - Dec 2023	125	Adults	Petersdorf & Beeson	[26]
6	Tsheten (2022)	Bhutan	Cross-sectional	Jan 2020 - Dec 2020	290	Mixed	Locally adapted	[27]
7	Kant (2023)	India	Mixed Method	May 2022 - Sep 2022	950	Medical	Not explicitly stated	[28]
8	Surong (2023)	India	Cross-sectional	Jan 2021 - Dec 2022	193	Children	Locally adapted	[29]
9	Nailli (2020)	India	Cross-sectional	Sep 2016 - Sep 2018	100	Children	Petersdorf & Beeson	[30]
10	Bandyopadhyay (2011)	India	Cohort	Sep 2008 - July 2009	164	Mixed	Petersdorf & Beeson	[31]
11	Das (2021)	India	Diagnostic Accuracy Test	Jun 2013 - May 2016	43	Adults	Petersdorf & Beeson	[32]
12	Singh (2021)	India	Cross-sectional	Sep 2017 - Sep 2020	1067	Mixed	Not explicitly stated	[33]
13	Lewin (2025)	India	Cross-sectional	Oct 2017 - Apr 2019	90	Children	Locally adapted	[34]
14	Dhangar (2025)	India	Cohort	Jan 2018 - Dec 2022	228	Adults	Locally adapted	[35]
15	Govindaraj (2024)	India	Cohort	Jul 2021 - Jul 2022	400	Adults	Not Explicitly Stated	[36]
16	Chrispal (2010)	India	Cohort	Jan 2007 - Jan 2008	398	Adults	Locally adapted	[37]
17	Ittyachen (2015)	India	Cross-sectional	Jan 2002 - Dec 2011	9739	Adults	Not Explicitly Stated	[38]
18	Abrahamsen (2013)	India	Cohort	Jul 2007 - Aug 2007	100	Adults	Not explicitly stated	[39]
19	Govindarajulu (2017)	India	Cohort	May 2016 - Oct 2016	120	Children	Locally adapted	[40]
20	Gandla (2021)	India	Cross-sectional	Aug 2018 - May 2020	NR	Children	Durack & Street	[41]
21	Reddy (2019)	India	Cohort	May 2012 - Apr 2016	69	Children	Not explicitly stated	[42]
22	Ramabhatta (2019)	India	Cohort	Mar 2017 - Feb 2018	24	Mixed	Petersdorf & Beeson	[43]
23	Kulkarni (2020)	India	Cross-sectional	NR	200	Mixed	Petersdorf & Beeson	[44]
24	Ilamparithi (2022)	India	Cross-sectional	Aug 2019 - Mar 2020	182	Children	Petersdorf & Beeson	[45]
25	Banode (2025)	India	Cohort	Jan 2013 - Nov 2014	80	NR	Petersdorf & Beeson	[46]
26	Pannu (2021)	India	Cohort	Jan 2018 - Dec 2019	152	Adults	Petersdorf & Beeson	[47]
27	Shankar (2014)	Nepal	Cross-sectional	Jun 2011 - Nov 2011	883	Adults	Not explicitly stated	[48]
28	Dhungana (2012)	Nepal	Cross-sectional	Jan 2010 - Jan 2012	898	Mixed	Locally adapted	[49]
29	Murdoch (2004)	Nepal	Cohort	Jan 2001 - Aug 2001	876	Adults	Not explicitly stated	[50]
30	Kattel (2019)	Nepal	Cohort	Jan 2013 - Dec 2014	245	Adults	Not explicitly stated	[51]
31	Pokhrel (2020)	Nepal	Case-Control	Jun 2016 - Jul 2016	81	Adults	Durack & Street	[52]
32	Jabeen (2022)	Pakistan	Cross-sectional	Sep 2018 - Feb 2019	120	Adults	Locally adapted	[53]
33	Khattak (2011)	Pakistan	Cohort	Mar 2008 - Mar 2009	100	Adults	Petersdorf & Beeson	[54]
34	Mahmood (2013)	Pakistan	Cross-sectional	Jan 2006 - Dec 2011	205	Mixed	Durack & Street	[55]
35	Akhtar (2022)	Pakistan	Cohort	Jan 2018 - Dec 2021	412	Mixed	Durack & Street	[56]
36	Premathilaka (2023)	Sri Lanka	Cross-sectional	Jan 2015 - Jan 2020	100	Mixed	Petersdorf & Benson	[5]
37	Bodinayake (2023)	Sri Lanka	Cohort	Jun 2012 - May 2013	976	Mixed	Durack & Street	[57]

Studies are presented by country in alphabetical order; ‘NR’ indicates Not Reported. The majority of included studies were conducted in tertiary hospital (TH) settings; study setting was not reported in one study. To reduce redundancy, the setting column was omitted. Some studies used the terms FUO and PUO interchangeably. “Locally adapted definition” means case inclusion based on author-defined criteria adapted to local clinical or diagnostic practices.

**Table 2:** Risk of bias assessment of included studies

Ref.	Author (Year)	Design, Tool	Overall risk of bias*	Key concerns
[22]	Haidar (2018)	Cross-sectional, JBI	Moderate	Lack of confounding control, variability in diagnostic confirmation, and single-center design.
[23]	Haque (2025)	Cross-sectional, JBI	Moderate	Convenience sampling, single-center design, lack of confounding control, and variability in diagnostic confirmation.
[24]	Rahimi (2016)	Cross-sectional, JBI	Moderate	Small sample size (n = 33), single-center design, and a highly specialized patient population (84.8% diabetic).
[25]	Haque (2025)	Cohort, JBI	Moderate	Single-center design, modest sample size (n = 80), lack of confounding control, and absence of a comparison group.
[26]	Bhuiyan (2024)	Cross-sectional, JBI	Moderate	Methodological constraints lead to unclear and potentially inconsistent outcome ascertainment, no identification or control of confounding factors, and limited interpretability of etiological distribution.
[27]	Tsheten (2022)	Cross-sectional, JBI	Moderate	The single-center, convenience sampling method failed to identify and measure key confounders.
[28]	Kant (2023)	Mixed Method, JBI	High	Unclear, potentially non-representative sampling method; No control for important confounders (e.g., setting, region); Outcomes based on self-report.
[29]	Surong (2023)	Cross-sectional, JBI	Moderate	Single-center tertiary hospital design may limit generalizability; potential confounding factors (e.g., prior treatment) were not analyzed.
[30]	Nailli (2020)	Cross-sectional, JBI	Moderate	There was a lack of confounder assessment and limited statistical analysis, despite clear case definitions and robust outcome ascertainment.
[31]	Bandyopadhyay (2011)	Cohort, JBI	Moderate	High risk of outcome misclassification due to reliance on therapeutic trials for tuberculosis diagnosis, combined with an unexplained loss of follow-up and a lack of strategies to address attrition-related bias.
[32]	Das (2021)	Diagnostic Accuracy,QUADAS 2	High	There is a lack of independence between the index test and reference standard, and there are substantial exclusions in patient flow.
[33]	Singh (2021)	Cross-sectional, JBI	Moderate	There was a failure to identify and measure key confounders.
[34]	Lewin (2025)	Cross-sectional, JBI	Moderate	Convenience sampling and single-center design. Lack of statistical adjustment for identified confounders. Prior antibiotic use in most patients may have obscured etiological diagnoses.
[35]	Dhangar (2025)	Cohort, JBI	Moderate	Lack of confounding control, high and unexplained loss to follow-up, and unclear baseline outcome status.
[36]	Govindaraj (2024)	Cohort, JBI	Moderate	Absence of inferential and multivariable analyses despite comprehensive confounder data collection and unclear reporting of attrition
[37]	Chrispal (2010)	Cohort, JBI	Low	The study employed standardized diagnostic approaches and systematic evaluation, minimizing selection and measurement bias.
[38]	Ittyachen (2015)	Cross-sectional, JBI	Moderate	"Presumed viral fever" diagnoses (lacking confirmatory testing), retrospective design, single-center setting, and lack of confounding control.
[39]	Abrahamsen (2013)	Cohort, JBI	Moderate	The etiological ascertainment was incomplete due to pending diagnostic results at discharge, and the follow-up was limited to the hospitalization period.
[40]	Govindarajulu (2017)	Cohort, JBI	Moderate	Lack of confounder assessment and absence of analytical adjustment, despite low attrition and appropriate follow-up for etiological diagnosis.
[41]	Gandla (2021)	Cross-sectional, JBI	Moderate	Variability in diagnostic confirmation, lack of confounding control, and single-center design.
[42]	Reddy (2019)	Cohort, JBI	Moderate	The study has a single-center design, potential selection bias (tertiary care setting), lack of confounding control, and unclear follow-up.
[43]	Ramabhatta (2019)	Cohort, JBI	Moderate	Very small sample size (n = 24), single-center design, lack of identification or control for confounders, and incomplete reporting of follow-up.
[44]	Kulkarni (2020)	Cross-sectional, JBI	Moderate	No confounding factor identification and adjustment, Single-center, convenience sampling, Exclusion of non-infectious causes limits etiological spectrum analysis.
[45]	Ilamparithi (2022)	Cross-sectional, JBI	Moderate	Variability in diagnostic confirmation, lack of confounding control, and single-center design.
[46]	Banode (2025)	Cohort, JBI	High	There is a high risk of outcome misclassification due to unclear diagnostic criteria and reliance on therapeutic trials for tuberculosis, with additional bias arising from unexplained loss to follow-up and a lack of methods to address confounding or missing data.
[47]	Pannu (2021)	Cohort, JBI	Moderate	There was a lack of identification and adjustment for potential confounding factors in the analysis of PUO etiology.
[48]	Shankar (2014)	Cross-sectional, JBI	Moderate	Reliance on clinical diagnoses for many cases, single-center, hospital-based data with no adjustment for potential confounding factors.
[49]	Dhungana (2012)	Cross-sectional, JBI	High	Reliance on clinical diagnosis without consistent lab confirmation, lack of control for confounders, and potential underestimation of true disease burden
[50]	Murdoch (2004)	Cohort, JBI	Low	The study had limited follow-up serology, with only 13% of participants providing convalescent samples, which may lead to an underestimation of some infections due to prior antibiotic use, and it was conducted at a single center.
[51]	Kattel (2019)	Cohort, JBI	Moderate	Lack of statistical adjustment for confounders. Reliance on self-reported exposure data. Single-center design, limiting generalizability.
[52]	Pokhrel (2020)	Case-Control, JBI	Moderate	Use of convenience sampling, complete lack of control for confounding factors, and limited etiological data.
[53]	Jabeen (2022)	Cross-sectional, JBI	Moderate	There is potential diagnostic variability (some diagnoses are not gold-standard confirmed), a single-center design, and a lack of confounding control.
[54]	Khattak (2011)	Cohort, JBI	Moderate	Single center, Lack of comparative analysis or adjustment for confounders, Limited post-discharge follow-up.
[55]	Mahmood (2013)	Cross-sectional, JBI	Moderate	Lack of analytical adjustment for confounders. No follow-up data for undiagnosed cases (11.7% remained undiagnosed).
[56]	Akhtar (2022)	Cohort, JBI	Moderate	There was no control for identified confounders, and the duration and completeness of the follow-up were unclear.
[5]	Premathilaka (2023)	Cross-sectional, JBI	Moderate	The retrospective design, single-center setting, and lack of control for confounding in cost comparisons pose significant challenges. The high rate of empirical antibiotic use is a notable contextual factor.
[57]	Bodinayake (2023)	Cohort, JBI	Moderate	Absence of multivariable adjustment for identified confounders and unclear management of loss to follow-up

**Table 3:** Diagnostic Approaches and Etiological Spectrum Reported Across Studies

Ref.	Author (Year)	Diagnostic Modalities Used	Final	Etiological Category				Undiagnosed (%)	Most Common Specific Etiology
			Diagnosis Achieved (%)	Infectious (%)	NIIDs/Auto-immune (%)	Malignancies (%)	Miscellaneous (%)		
[22]	Haidar (2018)	Basic laboratory, serology	100	43.06	25	20.83	6.94	NR	EP - TB
[23]	Haque (2025)	Basic Laboratory, Serology, Biopsy, Imaging	100	100	NR	NR	NR	NR	UTI
[24]	Rahimi (2016)	Basic Laboratory, Serology, Imaging, Histopathology, Hematology, Molecular Test	97	60.6	3	27.3	6.1	3	NHL
[25]	Haque (2025)	Basic Laboratory, Serology, Imaging, Biopsy	100	63.3	21.2	12.5	NR	NR	TB
[26]	Bhuiyan (2024)	Basic laboratory, serology, imaging	83	48	24	16	12	17	TB
[27]	Tsheten (2022)	Basic laboratory, Serology, Microbiology, Imaging	100	100	NR	NR	NR	NR	RTI
[29]	Surong (2023)	Basic laboratory, serology, imaging	90	62.5	10	9.5	8	10	RTI
[30]	Nailli (2020)	Basic laboratory, Serology, Microbiology, Imaging	84	38	22	24	NR	16	ALL
[31]	Bandyopadhyay (2011)	Basic laboratory, serology, imaging, and molecular tests	88	58.53	11	22	NR	12.2	TB
[32]	Das (2021)	Basic laboratory, serology, imaging, biopsy	74	23.3	37.2	9.3	4.7	NR	TB
[33]	Singh (2021)	Serology, molecular tests	90.9	90.9	NR	NR	NR	9.1	Scrub Typhu
[34]	Lewin (2025)	Basic laboratory, serology, microbiology, histology, imaging, and molecular tests	76.7	51.1	15.6	10	NC	23.3	Rickettsial Infection
[35]	Dhangar (2025)	Clinical parameters and diagnostic investigation	NC	NR	NR	NR	NR	36.5	Infectious
[36]	Govindaraj (2024)	Basic laboratory, serology, molecular testing	81	81	NR	NR	NR	19	Dengue
[37]	Chrispal (2010)	Basic laboratory, serology, imaging	92	NC	NC	NC	NC	8.0	Scrub Typhu
[38]	Ittyachen (2015)	Culture, Serology	99.4	99.4	NR	NR	NR	0.6	Presumed viral fever
[39]	Abrahamsen (2013)	Basic laboratory, serology, imaging	86	71	9	6	NR	13%	TB
[40]	Govindarajulu (2017)	Basic laboratory, serology, imaging	96.6	69.1	5	16.7	5.8	3.4	Enteric Feve
[41]	Gandla (2021)	Basic laboratory, serology	93.3	75.3	NR	10	NR	6.7	Enteric Feve
[42]	Reddy (2019)	Basic laboratory, serology, histology, imaging	81.2	31.8	14.5	20.2	14.5	18.8	TB
[43]	Ramabhatta (2019)	Basic laboratory, serology, imaging, and molecular tests	91.5	70.8	4.1	8.3	8.3	8.3	TB
[44]	Kulkarni (2020)	Basic laboratory, serology	84.5	84.5	NR	NR	NR	15.5	Dengue
[45]	Illamparithi (2022)	Basic laboratory, serology, imaging	83.6	37.4	22	24.2	NR	16.5	Mix
[46]	Banode (2025)	Basic laboratory, serology, imaging	82.50	48.75	12.5	11.2	10	17.50	TB
[47]	Pannu (2021)	Basic laboratory, serology, imaging	86.6	43.4	19.7	21.5	2	12.5	TB
[48]	Shankar (2014)	NR	NC	NC	NC	NC	NC	NC	RTI
[49]	Dhungana (2012)	NC	NC	NC	NC	NC	NC	NC	Infective exacerbatio of COPD
[50]	Murdoch (2004)	Microbiology, Serology	37	NC	NR	NR	NR	NC	Salmonella enterica serotype typ & paratyphi
[51]	Kattel (2019)	Basic laboratory, serology	82	82	NR	NR	NR	18	Pneumonia
[52]	Pokhrel (2020)	Microbiology Serology	38.1	38.1	NR	NR	NR	61.9	Enteric Feve
[53]	Jabeen (2022)	Bone Marrow Biopsy	88.3	30.0	NC	NC	NC	11.7	Fever due to reactive changes
[54]	Khattak (2011)	Basic laboratory, serology, microbiology, histology, imaging	83	57	12	10	3	17	TB
[55]	Mahmood (2013)	Basic laboratory, serology, microbiology, imaging	83.4	48.8	12.7	12.7	3.4	11.7	TB
[56]	Akhtar (2022)	Basic laboratory, serology, imaging, and molecular tests	93.2	47.1	21.8	23.1	1.2	6.8	TB
[5]	Premathilaka (2023)	Serology, microbiology, histopathology, imaging, and molecular tests	65	72.31	20	7.7	1.53	35	EP-TB
[57]	Bodinayake (2023)	Basic laboratory, serology, virology, and molecular tests	67.6	Viral: 54.7 Bacterial: 14.1	NR	NR	NR	32.5	Dengue

Studies reporting available information for these outcomes are included in this synthesis (N < 37). Abbreviations: NC: Mentioned but unclear; NR: Not mentioned at all; EP-TB: Extrapulmonary Tuberculosis; NIIDs: Non-Infectious Inflammatory Diseases; UTI: Urinary Tract Infection; RTI: Respiratory Tract Infection.

**Table 4:** Summary of Reported Diagnostic and Systemic Barriers to PUO Management

Category	Key Barriers Reported	Representative Studies
Infrastructural/Technical	Limited laboratory capacity; absence of advanced imaging (PET-CT, MRI); lack of molecular diagnostics; long turnaround times for cultures; inadequate funding	Kant [28], Chrispal 2010 [37], Premathilaka 2023 [5], Ittyachen 2015 [38], Govindaraj 2024 [36], multiple others
Knowledge/Protocol- Based	Lack of standardized diagnostic algorithms; over-reliance on empirical treatment; limited training/knowledge; inconsistent clinical protocols	Kant 2023 [28], Surong 2023 [29], Dhangar 2025 [35], Akhtar 2022 [56]
Patient-Systemic	Late presentation, prior antibiotic use, socioeconomic constraints, poor healthcare-seeking behavior, loss to follow-up	Chrispal 2010 [37], Dhungana 2012 [49], Murdoch 2004 [50], multiple studies
Other	Changing epidemiological landscape, antimicrobial resistance, geographical factors, gender discrimination in care-seeking	Tsheten 2022 [27], Haque 2025 [23], Bhuiyan 2024 [26]

**Table 5:** Summary of Reported Management Strategies for PUO

Management Strategy	Description	Representative Studies (Author, Year)
Empirical Antimicrobial Therapy	Use of broad-spectrum antibiotics (e.g., cephalosporins, doxycycline) prior to definitive diagnosis.	Kant 2023 [28]; Premathilaka 2023 [5]; Kattel 2019 [51]
Diagnostic-Therapeutic Trial	Administering treatment (e.g., ATT) for a suspected condition and using clinical response as a diagnostic clue.	Bandyopadhyay 2011 [31]; Banode 2025 [46]; Pannu 2021 [47]
Specialty Referral & Targeted Treatment	Referring patients to specialized departments for definitive management of NIIDs or malignancies.	Reddy 2019 [42]; Tsheten 2022 [27]
Protocol-Driven Evaluation	Adopting a stepwise, algorithm-based approach to investigation and therapy based on clinical clues.	Akhtar 2022 [56]; Surong 2023 [29]; Bandyopadhyay 2011 [31]

## Data Availability

All data generated or analyzed are included in this article and its supplementary files. Supporting datasets, including extraction and quality assessments, are available from the corresponding author upon reasonable request. The protocol is registered in PROSPERO (CRD420251170142).

## Box 1. Summary of Literature Search Strategy

**Table:**

Databases searched	PubMed/MEDLINE, Google Scholar, Embase, Scopus, Web of Science, Index Medicus for the South-East Asia Region (IMSEAR) Other sources: Backward/forward citation tracking
Search concepts	- Pyrexia of unknown origin (PUO) / fever of unknown origin (FUO) - South Asian countries (India, Nepal, Pakistan, Bangladesh, Sri Lanka, Bhutan, Maldives, and Afghanistan)
Search terms	Controlled vocabulary (e.g., MeSH,) and free-text keywords including “Pyrexia of Unknown Origin,” “Fever of Unknown Origin,” “PUO,” “FUO,” and synonyms such as “undifferentiated fever”
Limits applied	Language: English Publication period: 2000 onward Population: Humans

## Abbreviations

AMR: Antimicrobial Resistance
PUO: Pyrexia of Unknown Origin
FUO: Fever of Unknown Origin
NIIDs: Non-Infectious Inflammatory Diseases
PDCs: Potential Diagnostic Clues

## References

[1] Fernandez C, Beeching NJ. Pyrexia of unknown origin. Clin Med. 2018;18:170. 10.7861/CLINMEDICINE.18-2-170. PMC6303444 29626024

[2] PETERSDORF RG, BEESON PB. FEVER OF UNEXPLAINED ORIGIN: REPORT ON 100 CASES. Medicine. 1961;40. 10.1097/00005792-196102000-00001 13734791

[3] Bleeker-Rovers CP, Vos FJ, De Kleijn EMHA, Mudde AH, Dofferhoff TSM, Richter C, A prospective multicenter study on fever of unknown origin: The yield of a structured diagnostic protocol. Medicine. 2007;86:26–38. 10.1097/MD.0B013E31802FE858. 17220753

[4] Beresford RW, Gosbell IB. Pyrexia of unknown origin: causes, investigation and management. Intern Med J. 2016;46:1011–6. 10.1111/IMJ.13180. ;WGROUP:STRING:PUBLICATION. 27633467

[5] Premathilaka R, Darshana T, Ekanayake C, Chathurangani KC, Mendis I, Perinparajah S, Pyrexia of unknown origin (PUO) and the cost of care in a tertiary care institute in Sri Lanka. BMC Health Serv Res. 2023;23:177. 10.1186/S12913-023-09169-1. 36810045 PMC9945736

[6] Cunha BA, Lortholary O, Cunha CB. Fever of unknown origin: A clinical approach. Am J Med. 2015;128:1138. 10.1016/j.amjmed.2015.06.001.. e1–1138.e15. 26093175

[7] Fusco FM, Pisapia R, Nardiello S, Cicala SD, Gaeta GB, Brancaccio G. Fever of unknown origin (FUO): which are the factors influencing the final diagnosis? A 2005–2015 systematic review. BMC Infect Dis. 2019;19:653. 10.1186/S12879-019-4285-8. 31331269 PMC6647059

[8] Wright WF, Yenokyan G, Auwaerter PG. Geographic Influence Upon Noninfectious Diseases Accounting for Fever of Unknown Origin: A Systematic Review and Meta-Analysis. Open Forum Infect Dis. 2022;9:ofac396. 10.1093/OFID/OFAC396. 36004312 PMC9394765

[9] Wright WF, Yenokyan G, Auwaerter PG. Geographic Influence Upon Noninfectious Diseases Accounting for Fever of Unknown Origin: A Systematic Review and Meta-Analysis. Open Forum Infect Dis. 2022;9:ofac396. 10.1093/OFID/OFAC396. 36004312 PMC9394765

[10] Siegel KR, Patel SA, Ali MK. Non-communicable diseases in South Asia: contemporary perspectives. Br Med Bull. 2014;111:31. 10.1093/BMB/LDU018. 25190759 PMC4416117

[11] Vicziany M. The Modernisation of South Asia’s Disease Burden: 1950 to 2021. South Asia. J South Asia Stud. 2021;44:1114–30. 10.1080/00856401.2021.2004002. ;SUBPAGE:STRING:FULL.

[12] Ilona Brown NA, Finnigan. Fever of Unknown Origin. StatPearls Publishing. 2023. https://www.ncbi.nlm.nih.gov/books/NBK532265/. Accessed 26 Dec 2025. 30335298

[13] Barathan M. From fever to action: diagnosis, treatment, and prevention of acute undifferentiated febrile illnesses. Pathog Dis. 2024;82. 10.1093/FEMSPD/FTAE006. PMC11067964 38614961

[14] Kakkar AK, Shafiq N, Singh G, Ray P, Gautam V, Agarwal R, Antimicrobial Stewardship Programs in Resource Constrained Environments: Understanding and Addressing the Need of the Systems. Front Public Health. 2020;8:502288. 10.3389/FPUBH.2020.00140/BIBTEX. PMC7198767 32411647

[15] Wright WF, Stelmash L, Betrains A, Mulders-Manders CM, Rovers CP, Vanderschueren S, Recommendations for Updating Fever and Inflammation of Unknown Origin From a Modified Delphi Consensus Panel. Open Forum Infect Dis. 2024;11:ofae298. 10.1093/OFID/OFAE298. 38966848 PMC11222709

[16] Wright WF, Auwaerter PG. Fever and Fever of Unknown Origin: Review, Recent Advances, and Lingering Dogma. Open Forum Infect Dis. 2020;7:ofaa132. 10.1093/OFID/OFAA132. 32462043 PMC7237822

[17] Durack DT, Street AC. Fever of unknown origin–reexamined and redefined. Curr Clin Top Infect Dis. 1991;11:35–51. 1651090

[18] Covidence systematic review software. Veritas Health Innovation.

[19] Munn Z, Hugh Barker T, Moola S, Tufanaru C, Stern C, McArthur A, Methodological quality of case series studies: an introduction to the JBI critical appraisal tool. JBI Evid Synth. 2020;18:2127–33. 10.11124/JBISRIR-D-19-00099. 33038125

[20] Moola S, Munn Z, Tufunaru C, Aromataris E, Sears K, Sfetc R Systematic reviews of Aetiology and risk. In: Aromataris E MZ, editor. JBI Manual for Evidence Synthesis. 2024th edition. JBI; 2020. 10.46658/JBIMES-24-06

[21] Whiting PF, Rutjes AWS, Westwood ME, Mallett S, Deeks JJ, Reitsma JB, QUADAS-2: A Revised Tool for the Quality Assessment of Diagnostic Accuracy Studies. Ann Intern Med. 2011;155:529–36. 10.7326/0003-4819-155-8-201110180-00009. 22007046

[22] Haidar MS. Clinico-Pathological Evaluation of Fever More Than Three Weeks: A Cross Sectional Study in A Tertiary Care Hospital. Chattagram Maa-O-Shishu Hospital Medical College Journal. 2018;17 2 PG-6–13:6–13.

[23] Haque MS, Rahman MR, Ahmed JU. Clinical spectrum and aetiology of patients with acute febrile illness: experience at a tertiary level hospital in Dhaka, Bangladesh. BIRDEM Med J. 2025;15(2 PG–58–62):58–62.

[24] Rahimi MA, Ahmed AKMS, Hossain MD, Rahman MR, Ghosh SK, Nazneen S, Aetiology of Fever of Unknown Origin: One-Year Experience in a Tertiary Care Hospital of Bangladesh. BIRDEM Med J. 2016;6:7–11. 10.3329/birdem.v6i1.28402.

[25] Haque MS, Chakraborty R, Islam MT, Rahman MR, Ahmed JU. Clinical Profile and Etiology of Prolonged Pyrexia in Adults Attending a Tertiary Care Hospital in Bangladesh. SAS J Med. 2025;4:362–7.

[26] Bhuiyan ME. Fever of Unknown Origin at A Tertiary Care Teaching Hospital in Bangladesh. Insight. 2024;7:157–66.

[27] Tsheten T, Lhendup K, Dorji T, Wangdi K. Aetiologies and Risk Factors of Prolonged Fever Admission in Samtse Hospital, Bhutan, 2020. Int J Environ Res Public Health. 2022;19. 10.3390/ijerph19137859. PMC9266161 35805518

[28] Kant S, Tripathi P. A Questionnaire-based Survey on Treatments and Practices of Antibiotics in URTI and Fever of Unknown Origin in India. Indian J Clin Pract. 2023;33.

[29] Surong N, Joseph D. Clinicoetiological profile of pyrexia of unknown origin in children 1–12 years of age in a tertiary care centre. Int J Contemp Pediatr. 2023;10:813–8. 10.18203/2349-3291.IJCP20231483.

[30] Nalli RK, Velampalli S, Borigama N. A Prospective Study of Clinico-Aetiological Profile of Fever of Unknown Origin in Children Admitted. to a Tertiary Care Center; 2020.

[31] Bandyopadhyay D, Bandyopadhyay R, Paul R, Roy D. Etiological study of Fever of unknown origin in patients admitted to medicine ward of a teaching hospital of eastern India. J Glob Infect Dis. 2011;3:329–33. 10.4103/0974-777X.91052. 22223993 PMC3249985

[32] Das S, Sathyendra S, Hephzibah J, Karuppusami R, Gunasekaran K, Shanthly N, Utility of positron emission tomography-computed tomography in the evaluation of fever of unknown origin in a resource-limited tropical nation. World J Nucl Med. 2021;20:237–46. 10.4103/wjnm.WJNM_99_20. 34703391 PMC8488897

[33] Singh S, Patel SS, Sahu C, Ghoshal U. Seroprevalence trends of Scrub typhus among the febrile patients of Northern India: A prospective cross-sectional study. J Family Med Prim Care. 2021;10:2552–7. 10.4103/jfmpc.jfmpc_2392_20. 34568135 PMC8415648

[34] Lewin M, Mary T. Etiology of Prolonged Fever in Children Using a Protocol-based Approach from a Tertiary-level Hospital in India: A Descriptive Study. Pediatr Infect Dis. 2025;7:54–8.

[35] Dhangar P, Panda PK, Kant R, Gupta R, Dua R, Tiwari A, Evaluating fever of unknown origin definitions in a tertiary care setting: Implications for diagnostic criteria revision. World J Exp Med. 2025;15:101388. 40546669 10.5493/wjem.v15.i2.101388 PMC12019626

[36] Govindaraj V, Poonia D, Bhardwaj G, Balasubramani S, Michael B. Identifying the Probable Etiology of Acute Undifferentiated Fever through Inflammatory Markers. J Assoc Physicians India. 2024;72:13–6. 10.59556/japi.72.0523. 38881103

[37] Chrispal A, Boorugu H, Gopinath KG, Chandy S, Prakash JAJ, Thomas EM, Acute undifferentiated febrile illness in adult hospitalized patients: the disease spectrum and diagnostic predictors - an experience from a tertiary care hospital in South India. Trop Doct. 2010;40:230–4. 10.1258/TD.2010.100132. 20870680

[38] Ittyachen AM, Ramachandran R. Study of acute febrile illness: a 10-year descriptive study and a proposed algorithm from a tertiary care referral hospital in rural Kerala in Southern India. Trop Doct. 2015;45:114–7. 10.1177/0049475514566264. 25540169

[39] Abrahamsen SK, Haugen CN, Rupali P, Mathai D, Langeland N, Eide GE Fever in the tropics: aetiology and case-fatality-a prospective observational study in a tertiary care hospital in South India. BMC Infect Dis. 2013;13 1 PG-355:355. 23899336 10.1186/1471-2334-13-355 PMC3750507

[40] Govindarajulu S, Kalyanasundaram K, Pyarejan KS, Venkatasamy S. Clinical profile and etiological spectrum of fever of unknown origin in children aged 2 months to 12 years. Int J Contemp Pediatr. 2017;4(1 PG–62–7):62–7.

[41] Gandla N, Bharani SA, Shah TP. A study of etiology of fever of unknown origin in children aged 2 months to 18 years. Int J Contemp Pediatr. 2021;8 11 PG-1842:1842.

[42] Reddy PA, Reddy MS. Profile of fever of unknown origin in children and the role of investigation: an observational study. J Microbiol Infect Dis. 2019;9:137–43. 04 PG-137–143.

[43] Ramabhatta S, Kariyappa P, Sanikam H, Kumar SU, Pujar P, Kittagaly P. Clinico-aetiological profile of fever of unknown origin (FUO) in children in a single centre. Sri Lanka J Child Health. 2019;48 2 PG-.

[44] Kulkarni S, Chillarge C. A prospective study on the microbial profile of pyrexia of unknown origin from inpatients of tertiary care hospital in. North Karnataka. PG-; 2020.

[45] Ilamparithi P. Etiological Profile Of Fever Of Unknown Origin In Children Between 1 Month To 12 Years Admitted In An Urban Referral Centre. Int J. 2022;5 6 PG-506:506.

[46] Banode DR, Nandkishor R. Sirkanungo Dr padmani A] DrCB, Roy DrA. A Prospective Study of Cases of Pyrexia of Unknown Origin Admitted At A Tertiary Care Institute With Reference To Their Etiology and Outcome. Int J Life Sci. 2025;14. 10.69605/ijlbpr.

[47] Pannu AK, Golla R, Kumari S, Suri V, Gupta P, Kumar R. Aetiology of pyrexia of unknown origin in north India. Trop Doct. 2021;51:34–40. 10.1177/0049475520947907;WGROUP:STRING:PUBLICATION. 32807027

[48] Shankar L, Saikia PB, Kumar N, Khusraj D, Kumudini S, Ramsundar T, A study of pattern of acute febrile illnesses at COMS-TH, Bharatpur, Nepal. Asian Pac J Trop Dis. 2014;4(4 PG–297–300):297–300.

[49] Dhungana SP, Shrestha SK, Kashyap AK, Piryani RM, Acharya GP. The etiology of fever in patients presented at KIST Medical College, Teaching Hospital, Lalitpur, Nepal. Nepal Med Coll J. 2012;14(3 PG–241–243):241–3. 24047025

[50] Murdoch DR, Woods CW, Zimmerman MD, Dull PM, Belbase RH, Keenan AJ, The etiology of febrile illness in adults presenting to Patan hospital in Kathmandu, Nepal. Am J Trop Med Hyg. 2004;70:670–5. 6 PG-670–675. 15211012

[51] Kattel V, Agrawal Y, Pandey NK, Dahal S, Khanal B. Outcome OF Inpatient Acute Febrile Illness in A Referral Tropical Health Center in Nepal. J Trop Dis. 2019;7:PG.

[52] Pokharel S, Karki M, Acharya B, Marasini B, Arjyal A. Outbreak of acute undifferentiated febrile illness in Kathmandu, Nepal: clinical and epidemiological investigation. BMC Infect Dis. 2020;20:89. 10.1186/S12879-020-4803-8. 32000695 PMC6993335

[53] Jabeen S, Farooq M, Naeem S, Khan S, Farid M, Tasneem A, The role of bone marrow biopsy in patients with pyrexia of unknown origin. J Fatima Jinnah Med Univ. 2022;16:12–5.

[54] Khattak MI, Ishaq T, Amin S, ur Rehman S, Shabbir G. Pyrexia of unknown origin: Aetiologic frequency in a tertiary care hospital. Gomal J Med Sci. 2011;9:PG1–.

[55] Mahmood K, Akhtar T, Naeem M, Talib A, Haider I, Siraj-Us-Salikeen. Fever of unknown origin at a teritiary care teaching hospital in Pakistan. Southeast Asian J Trop Med Public Health. 2013;44:503–11. 24050083

[56] Akhtar W, Awan S, Ishaq U, Malik A, Malik J, Zaidi SMJ. Pyrexia of unknown origin and its aetiology in Pakistan. Trop Doct. 2022;52:567–71. 10.1177/00494755221096902. 35833343

[57] Bodinayake CK, Nagahawatte A, Devasiri V, Arachichi WK, Kurukulasooriya R, Sheng T, Comprehensive diagnostic testing identifies diverse aetiologies of acute febrile illness among hospitalised children and adults in Sri Lanka: a prospective cohort study. BMJ Public Health. 2023;1:PG1–. 10.1136/bmjph-2023-000073 PMC11812697 40017865

[58] Kant S, Tripathi P. A Questionnaire-based Survey on Treatments and Practices of Antibiotics in URTI and Fever of Unknown Origin in India. 2023;33.

[59] Wright WF, Yenokyan G, Auwaerter PG. Geographic Influence Upon Noninfectious Diseases Accounting for Fever of Unknown Origin: A Systematic Review and Meta-Analysis. Open Forum Infect Dis. 2022;9:ofac396. 10.1093/OFID/OFAC396. 36004312 PMC9394765

[60] Knockaert DC, Vanderschueren S, Blockmans D. Fever of unknown origin in adults: 40 years on. J Intern Med. 2003;253:263–75. 10.1046/J.1365-2796.2003.01120.X. 12603493

[61] Bleeker-Rovers CP, Vos FJ, De Kleijn EMHA, Mudde AH, Dofferhoff TSM, Richter C, A prospective multicenter study on fever of unknown origin: The yield of a structured diagnostic protocol. Medicine. 2007;86:26–38. 10.1097/MD.0B013E31802FE858. 17220753

[62] Sandherr M, Stemler J, Schalk E, Hattenhauer T, Hentrich M, Hertenstein B, 2024 update of the AGIHO guideline on diagnosis and empirical treatment of fever of unknown origin (FUO) in adult neutropenic patients with solid tumours and hematological malignancies. Lancet Reg Health - Europe. 2025;51:101214. 10.1016/j.lanepe.2025.101214. 39973942 PMC11836497

[63] Haidar G, Singh N. Fever of Unknown Origin. N Engl J Med. 2022;386:463–77. 10.1056/NEJMRA2111003. 35108471

[64] Divecha CA, Tullu MS, Karande S. Challenges in implementing an Antimicrobial Stewardship Program (ASP) in developing countries. J Postgrad Med. 2024;70:185. 10.4103/JPGM.JPGM_228_24. 38984523 PMC11722715

[65] Cox JA, Vlieghe E, Mendelson M, Wertheim H, Ndegwa L, Villegas MV, Antibiotic stewardship in low- and middle-income countries: the same but different? Clin Microbiol Infect. 2017;23:812–8. 10.1016/J.CMI.2017.07.010. 28712667

[66] Souweine B, Veber B, Bedos JP, Gachot B, Dombret MC, Regnier B Diagnostic accuracy of protected specimen brush and bronchoalveolar lavage in nosocomial pneumonia: Impact of previous antimicrobial treatments. Crit Care Med. 1998;26. 10.1097/00003246-199802000-00017 9468159

[67] Hirani R, Podder D, Stala O, Mohebpour R, Tiwari RK, Etienne M. Strategies to Reduce Hospital Length of Stay: Evidence and Challenges. Med (B Aires). 2025;61:922. 10.3390/MEDICINA61050922. PMC12112870 40428880

[68] Alharbi TAF, Rababa M, Alsuwayl H, Alsubail A, Alenizi WS. Diagnostic Challenges and Patient Safety: The Critical Role of Accuracy – A Systematic Review. J Multidiscip Healthc. 2025;18:3051. 10.2147/JMDH.S512254. 40470160 PMC12134007

[69] Pannu AK. Approach to pyrexia of unknown origin in low- and middle-income countries. Trop Doct. 2025;55:364–7. 10.1177/00494755251333964. 40223284

[70] Tinker NJ, Foster RA, Webb BJ, Haydoura S, Buckel WR, Stenehjem EA. Interventions to optimize antimicrobial stewardship. Antimicrob Stewardship Healthc Epidemiology: ASHE. 2021;1:e46. 10.1017/ASH.2021.210. 36168471 PMC9495515

[71] Baryakova TH, Pogostin BH, Langer R, McHugh KJ. Overcoming barriers to patient adherence: the case for developing innovative drug delivery systems. Nat Rev Drug Discov. 2023;22:387. 10.1038/S41573-023-00670-0. 36973491 PMC10041531

[72] Obhai G, Obhai G. From Self-Medication to Antimicrobial Resistance: Socioeconomic Realities and Public Health Implications in Kibera, Nairobi. Open J Prev Med. 2025;15:45–69. 10.4236/OJPM.2025.154004.

